# Hospital and Institutional News

**Published:** 1920-10-02

**Authors:** 


### HOSPITAL AND INSTITUTIONAL NEWS.

### CAUSES OF BLINDNESS.

The Minister of Health has appointed a Committee to investigate and report on the causes of blindness, and to suggest measures for prevention. The following have been asked to serve:—Mr. G. H. Roberts, M.P., chairman; Mr. Stephen Walsh, M.P.; Mr. N. Bishop Harman, M.B., F.R.C.S.; Dr. J. B. Lawford, M.D., F.R.C.S.; Mr. G. F. Mowatt; Mrs. Wilton Phipps; Mr. J. H. Parsons, C.B.E., F.R.C.S., representing the Royal College of Surgeons; Dr. J. Taylor, M.D., F.R.C.P., representing the Royal College of Physicians; Mr. J. C. Bridge, F.R.C.S., representing the Home Office; Dr. A. Eicholz, C.B.E., M.D., representing the Board of Education; Mr. J. S. Nicholson, representing the Ministry of Labour; Mr. W. M. Stone, representing the Scottish Office; Mr. E. D. Macgregor, representing the Ministry of Health; and a representative of the Medical Research Council (to be appointed later). Dr. R. A. Farrar and Mr. P. N. R. Butcher will act as joint secretaries to the committee. It would have been advantageous to include a member who is specially acquainted with industrial blindness, such as miners' nystagmus which costs the country over a million pounds sterling each year, as well as the health and happiness of a multitude of men.

### NATIONAL ASSOCIATION FOR THE PREVENTION OF TUBERCULOSIS.

As previously announced in our columns, the eighth annual conference of the above Association will be held, with the co-operation of the municipal authorities, at St. George's Hall, Liverpool, from October 7 to 10 inclusive. Further details are now to hand, and the subjects for discussion are: (1) Review of methods of prevention and treatment advocated from time to time, the extent to which they have been followed, and the results obtained; (2) practical difficulties in connection with the carrying out of tuberculosis schemes and the best measures to overcome them; (3) milk as a source of infection and the preventive measures to be employed. Amongst those who are expected to take part are Sir Robert Philip, Professor Moore, and Professor Adami. We miss in the list of names given any mention of those interested in what is now known as Labour. As the times show abundantly, this is a serious omission. Tuberculosis is, before all else, a sociological problem, and it is only by the active co-operation of all bodies, and particularly of those who have most knowledge of working-class needs, that success in prevention or cure can be assured. It seems strange to read that this conference is only the eighth held by this familiar Association, but the hiatus of the war is responsible.

### REGISTRATION OF BLIND CHARITIES.

We are glad to see that the War Charities Act, 1916, which apparently still stands, although the war is over, has, according to a Ministry of Health circular, been applied to charities for the blind, and it is now made illegal for any public appeal for donations to be issued unless the charity is registered and its governing body has approved the appeal in writing. An offence is punishable on summary conviction by a fine of not more than £100. There are one or two exceptions to the rule as to the written approval of the governing body, but the general effect will be that several societies which have been rather more than suspect in the past will now be snuffed out, and only those will remain which are registered by the Ministry of Health and prepare their accounts on the lines laid down by that department.

### WHAT IS AN ADMINISTRATOR?

The post of "Administrator" now vacant at the Radcliffe Infirmary, Oxford, is one which has been suggested by the experience gained of watching the best administration in all kinds of hospitals during the war. There is no desire to start a "medical superintendent," as the system whereby the medical needs and policy of a hospital are communicated to the committee as the united opinion of the whole staff is preferred. The business of the hospital, it is considered, should be managed by skilled men of rather different training and outlook. We are informed that a man is wanted who will take the initiative in working out hospital problems and see things whole. He will work in conjunction with an executive committee of five lay members who report monthly to the Committee of Management, and they in turn report to the Court of Governors quarterly. He will direct all the departments of the hospital, save those that come under the matron's care, and it is hoped will inspire the whole place. The salary, fixed at £600, is a minimum, and it is hoped that a satisfactory future may be arranged for any man who is found suitable for the position. The hospital is a thoroughly equipped general hospital, with a new extension for preventive work in progress on a site about two miles away. Moreover, there is great need to co-ordinate the work of several small cottage hospitals in the area, and to link them up in work and enthusiasm with the central hospital. The work, therefore, would be on rather new lines and extremely interesting.

### THE EXTENSIONS AT CHESTERFIELD.

Towards the appeal for £50,000 on behalf of the Chesterfield Royal Hospital extensions about half has been raised, and a satisfactory result has been obtained by the co-operation of employers and their workpeople. We understand that the employers have agreed to give rather more than twice the sum raised by the subscriptions of the men in 1919. This contribution is towards the capital cost of the extensions. In respect of the maintenance, the workpeople have agreed to increase their subscriptions from $1\frac{1}{2}$d. to $2\frac{1}{2}$d. per head per week, and the employers have consented to add $33\frac{1}{3}$ per cent. to these increased subscriptions. A

public appeal is now being directed to other classes of citizens, whom we hope it may be possible to organise on equally comprehensive lines.

### ILL-NOURISHED CHILDREN.

Some remarkable facts about malnutrition of children are given by Dr. Hyslop Thomson in his 1919 report as county medical officer of health for Hertfordshire. Some degree of malnutrition was found in 903 children, compared with 167 the previous year, the percentages being 6.6, compared with 1.3 in 1918 and 1.6 in 1917. This shows a considerable increase, and is no doubt due to the fact that as a result of the greatly decreased amount of female labour the family income is less, while the cost of living has increased. During school medical inspection it has been observed that the child who suffers from some degree of malnutrition is not always the dirty and apparently neglected one, but is frequently clean and well cared for. Dr. Ewing, for the Buntingford and Hadham district schools, reports that children are better clothed and fed, but that too many of them appear to be anæmic, and this he regards as probably due to a deficient vegetable diet. In the course of medical inspection one is frequently impressed by the pallor of well-nourished children in rural districts. Such pallor is no doubt due in a large measure to errors in diet and the lack of efficient ventilation of the sleeping rooms. In regard to teeth, the observation is made that the poorest type of child has frequently the best teeth.

### INDIAN LEPERS ACT.

With reference to our recent note concerning the more hopeful outlook in leprosy treatment and the isolation of the active principle of chaulmoogra oil, it is interesting to note the recent legislative developments in India. It has long been evident to all engaged in Indian administration that the provisions of the law relating to compulsory segregation of the pauper lepers are inadequate, and a Bill to amend the Leper Act has been introduced in the Imperial Council to make good the deficiency. This question is of profound importance to the whole Indian community, who now run great risks owing to the number of lepers who are at large. The question of segregating lepers belonging to other classes is more difficult, and does not now arise. In India as a whole there are .69 per cent. of the population suffering from leprosy. In some districts, among them Simla, Almora, Mauthum, and certain parts of Burma, the percentage is nearly twice as great. The important conference on the leper problem held in Calcutta placed it on record that, in the opinion of those present, the disease could be stamped out in India if all lepers were segregated, but as this does not appear to be practicable at this time it strongly urges that the first step to be taken in this direction is the segregation of all pauper lepers. That, apparently, will become possible if the said Bill becomes law. The Indian Press is drawing attention to the fact that the person suffering from leprosy is not so hapless as was once believed. One of the findings of the Calcutta Conference showed that under the modern methods of treatment 72 per cent. show marked improvement, even the most advanced cases, after quite a short period of treatment.

### LIVERPOOL AND SANATORIA.

The accommodation of tuberculosis cases in Liverpool is causing additional difficulty, because the Liverpool City Hospitals Committee has to vacate Park Hill, the site being wanted by the Dock Board. The Corporation has invited the help of the Liverpool Poor-Law Unions, and the Select Vestry has decided to lease Highfield Infirmary to the Corporation for two years, provided that the Corporation will treat all tuberculous patients for whom the Select Vestry would otherwise be responsible. Before the war, which interfered with the scheme, the Corporation had an ambitious programme for treating these cases at the new sanatorium at Fazakerley. The official opening of these buildings is timed to take place in October.

### MATERNITY WORK AT WOOLWICH.

The foundation-stone of the British Hospital for Mothers and Babies, Woolwich (National Training-school for District Midwives), will be laid by Her Royal Highness Princess Christian on Saturday, October 16, at 3 p.m. The object with which the institution was founded in 1905 was to improve and lengthen the training of midwives, and it received from its inception the warm support and encouragement of the late Dr. Cullingworth and other authorities. The work of the hospital has hitherto been carried out on a very small scale owing to lack of funds, and it is now refusing from twenty to thirty mothers every week. A three-acre freehold site has been purchased at Woolwich, and the committee have in hand sufficient money to provide one-third of the entire building. Donations are earnestly requested, to avoid the necessity of closing down the work before completion. They may be sent to The Lady Balfour of Burleigh, 47 Cadogan Square, S.W. 1, or to Miss Alice Gregory, Hon. Sec., British Hospital for Mothers and Babies, Woolwich. Chairman, Lieut.-Col. Sir Richard Temple, Bart., C.B., C.I.E.

### CHAPLAINS' SALARIES AND COST OF LIVING.

The Rev. T. C. Brothers, chaplain to the Abbeydore Board of Guardians, Herefordshire, lately applied for an increase of his salary of £30 a year, on the ground that it had not been raised for sixteen years. The Guardians offered him a further £5 a year, which he refused. The Poor Law Officers' Association, in support of the chaplain's request, reminded the Guardians that since July 1914 the cost of living had risen by 155 per cent., and that no class had been more affected thereby than the clergy. The Association asks for a bonus of £39 a year, in accordance with the scale for the Civil Service sanctioned for the Poor-Law service by the Ministry of Health. Owing to a technicality the matter will be raised again, when we hope that the chaplain's position will be much more sympathetically considered.

### THE RED CROSS CAMPAIGN.

The concluding meeting of the series of conferences of representatives of the British Red Cross Society and the Order of St. John of Jerusalem is, we are informed as we go to press, being held at the Guild Hall, Cambridge, on Friday, October 1, at 2 p.m., when representatives from Northamptonshire, Norfolk, Suffolk, Bedfordshire, Cambridgeshire, and Huntingdonshire assemble. Sir Arthur Stanley and Sir Napier Burnett are both speaking at this meeting, and we hope to refer to the proceedings in our next issue. With regard to the announcement of the co-operation of the League of Mercy and the Hospital Saturday Fund, we are authorised to state that an appropriate proportion of the proceeds of Our Day will be allocated to the London hospitals, who will no doubt gladly welcome this first intimation that they are to share in the fund which is one of the objects of the peace-time activities of the joint societies.

### THE RED CROSS AND ARMISTICE SUNDAY.

We have received an advance copy of the Appeal which will be issued shortly to all Churches, asking for all collections on Armistice Sunday, November 14. The Appeal bears the signature of Her Majesty Queen Alexandra, and His Royal Highness the Duke of Connaught, and it has been approved by the Archbishops, His Eminence the Cardinal, and by the Heads of the Free Churches. The money received will be devoted to several objects, amongst which may be cited: The care of sick and wounded ex-Service men; Maternity and Child Welfare; the upkeep of motor ambulances, some hundreds of which have been stationed throughout the country, and which are serving a most valuable purpose; and lastly, substantial grants to the civil hospitals in London and the provinces, which, especially in view of the approach of winter, it is important should be financially strengthened. The Joint Societies, at the request of the Churches, have arranged to share the sum collected with the Imperial War Relief Fund.

### THE MANCHESTER CONFERENCE.

An important conference took place at the Town Hall, Manchester, recently, during the tour of Sir Arthur Stanley and Sir Napier Burnett, when a large representative meeting of the British Red Cross Society and the Order of St. John of Jerusalem assembled to hear addresses by these gentleman. Sir Arthur Stanley informed the audience that at headquarters in London a great number of questions came before the executive body which they felt could not be answered without a visit to the different counties, and with his characteristic smile he remarked that one thing they learned during the war was that counties liked to manage their own business in their own way, and another that every county managed its business in a way different to the county next to it. The first and most decisive thing that they learned was that the counties were united against interference from London. It was the intention of himself and Sir Napier Burnett to extend their tour until they had met all the Red Cross representatives of all the counties in England and Wales. Scotland was looking after its own affairs, and Ireland, about which he would say nothing except that throughout the war some of the best and most loyal support came from that country, had sent a great deal of money, and was now asking for it back again, and he thought they honestly deserved it.

### THE V.A.D. SERVICE.

Sir Arthur Stanley explained the necessary combination of the Red Cross Society and the Order of St. John, and said that when he first had any acquaintance with the matter the two societies were so obviously engaged in fighting one another that they had very little time to do anything else. They quickly found that the position was impossible without combination, and when the Armistice came it was felt that it would be a thousand pities if the associations were to part company once more. This would be especially so because there was an enormous amount of work still to be done which must, and ought to be, done jointly. The peace-time programme has already been sketched in these columns. All this programme affected members of the Voluntary Aid Detachment, he especially pointed out, and referring to the ambulance service, stated that the motor ambulance has come into existence, so far as general use was concerned, absolutely since August 1914. They had a large number of ambulances, and they aimed at establishing a network of services throughout the whole of the country in such a way that no man, woman, or child who was sick or in any way injured would be more than ten or fifteen miles away from a motor ambulance, in other words, that if an accident occurred, within half an hour an ambulance should be on the road to taking the patient to the nearest hospital for treatment. Another way in which V.A.D. members could help was in giving aid to district nurses. With their ambulance service assisting he would like to see an idea carried out that in every small village, hamlet, and town throughout the country there should be some of their trained V.A.D.s.

### FALLACY OF RED CROSS MILLIONS.

Sir Arthur Stanley touched upon the question of finance, and said that he felt there was considerable misunderstanding in the matter. People had asked how it was that the Red Cross and the Order of St. John were begging for money in connection with Our Day when they had millions of pounds in London subscribed during the war. Sir Arthur said that if they really had millions he would retire from the business of public beggar, and would be only too delighted to do so. It was true that at the end of the war there was a considerable amount in hand, and one of their chief expenses, which was feeding prisoners in enemy countries, stopped almost immediately, but the money was given for the treatment of Service patients, and in respect of the two and a-quarter millions remaining over, under their Act of Parliament it was impossible to use the money for any other purpose.

### THE WAR SERVICE MEDAL.

The question of a medal for Red Cross workers was also dealt with. The Order of St. John had always had power to issue a medal, and he understood that they were doing so. In the case of the Red Cross, the matter had been referred to a Government committee, and a recommendation by that committee that the War Service medal should be given to all Red Cross workers in England as well as abroad was not accepted by the Government who, as yet, had not made up their minds what they were going to do. The matter had been held up in the hope that all the workers in the Red Cross and St. John would receive the Government medal, but as that had not been settled the Red Cross had decided to issue a medal. The lists were being drawn up and he hoped the distribution would begin very soon.

### SQUABBLES AT WELFARE CENTRES.

A scene at a meeting of the Llantwit Vadre Maternity and Child Welfare Centre has led to an inquiry by a sub-committee of the responsible authority, the Llantrisant Rural District Council. The facts, as stated in the report of the sub-committee, reveal a regrettable state of affairs. "The disturbance (says the report) was caused entirely by Nurse Allen, who accused Dr. Evans of having said that it was a mistake to have Trynant ladies on the committee, as they were not experienced enough. This Dr. Evans denied, and Nurse Allen thereupon called him a liar." This report was adopted at a meeting of the Llantrisant authority, but a motion that the nurse be called upon to apologise was defeated. It seems a thousand pities that differences between medical men and nurses should be of such frequent occurrence in South Wales. Not many weeks ago a medical officer publicly complained of the attitude of a matron and nursing staff, and said he would resign rather than have a rebellion against treatment he thought fit to order for patients. A few weeks ago public opinion was startled to learn of the difficulties at the Merthyr Poor-law Infirmary. A spirit of indiscipline, it appears, is abroad which needs to be checked.

### R.S.O. VERSUS VISITING STAFF.

Recently, at a house committee meeting, and further in public at a hospital festival, a proposal has been made that seems of far-reaching import to the medical profession. The situation is that at a hospital in an industrial district, staffed by general practitioners, assisted in the usual way by junior house surgeons, a representative of the workers in the staple industry has proposed that the surgical beds of the institution should be given over to a whole-time resident surgical officer, to be advertised for at a salary of £1,000 a year. It is argued that surgical emergencies frequently have to wait a long time before receiving adequate treatment—until, in fact, the honorary surgeon in question can be communicated with, which, in the forenoon, say, is a matter of difficulty. One well able to speak on that point gives the assurance that the workers would willingly subscribe more because of the extra benefit accruing to them. In such a locality accidents are frequent, and prompt treatment is therefore much to be desired. Presumably, employers would value the innovation as well. The medical side of the institution would be left as it is—that is, if the proposal, when put into effect, did not entail resignation of the honorary staff. Without pronouncing upon the matter, we might point out that the above arrangement is not without precedent. At the London and North-Western Railway's accident hospital at Crewe there is a surgical officer solely responsible for treatment of all admissions.

### KING EDWARD VII. HOSPITAL, CARDIFF.

The future of King Edward the Seventh Hospital, Cardiff, and the urgent need of development came up for discussion at a recent meeting of the Management Committee. An interim report was received from Colonel Donald J. Mackintosh, who had been asked to advise as to structural development, recommending that no further alterations be carried out, with the exception of re-arranging the accommodation for nurses, until a complete scheme has been prepared of all the requirements in relation to the University Medical School. An interim report also of the special committee appointed to consider the question of the location of a permanent nurses' home and of the future development of the hospital and its financial prospects was received. The committee, however, made no recommendation, suggesting further inquiries, and it was stated the Finance Committee would meet specially to consider the problems involved. It was reported at the meeting that the patients waiting admission to the wards numbered 1,153, and that the hospital's net overdraft amounted to £46,268—facts which themselves point to the gravity of the position both from the aspect of the public and the institution.

### FOOTBALL AND CHARITY.

The Fulham Football and Athletic Co. have sent a donation of ten guineas to the Royal Hospital and Home for Incurables, Putney, and the Mayor of Islington (Councillor E. H. King, J.P.) has forwarded to the memorial extension fund of the Great Northern Hospital a donation of fifty guineas from the Arsenal Football Club.

### THIS WEEK'S DRUG MARKET.

The market seems to be going from bad to worse, and business is quieter than ever. It is true that the outlook as regards the miners looks more hopeful, but until a definite decision ensuring peace, at any rate in the immediate future, is arrived at trade generally will be inactive. The trend of prices is the same as it has been for the past few months—namely, in a downward direction, although here and there one meets with the opposite tendency, as for instance in the case of eucalyptus oil. Among the articles that have moved downwards in price since our last report are cocaine, cream of tartar, citric acid, bromide of potassium, Japanese refined camphor, menthol, liquorice juice, tartaric acid and cod-liver oil. In synthetic drugs the situation is unchanged, and buyers are not at all disposed to increase their stocks.

